# Lack of Ephrin Receptor A1 Is a Favorable Independent Prognostic Factor in Clear Cell Renal Cell Carcinoma

**DOI:** 10.1371/journal.pone.0102262

**Published:** 2014-07-15

**Authors:** Marieta I. Toma, Kati Erdmann, Michael Diezel, Matthias Meinhardt, Stefan Zastrow, Susanne Fuessel, Manfred P. Wirth, Gustavo B. Baretton

**Affiliations:** 1 Institute of Pathology, University Hospital Carl Gustav Carus, TU Dresden, Dresden, Germany; 2 Department of Urology, University Hospital Carl Gustav Carus, TU Dresden, Dresden, Germany; Innsbruck Medical University, Austria

## Abstract

The EPH receptor tyrosine kinases and their cell-bound ligands, the ephrins, have been shown to be associated with cancer development and progression. In this study, mRNA and protein expression of the receptors EPHA1 and EPHA2 as well as of their ligand EFNA1 and their prognostic relevance in clear cell renal cell carcinoma was evaluated. Gene expression was measured in 75 cryo-preserved primary tumors and matched non-malignant renal specimens by quantitative PCR. Protein expression was analyzed by immunohistochemistry on tissue microarrays comprising non-malignant, primary tumors and metastatic renal tissues of 241 patients. Gene and protein expression of all three factors was altered in tumor specimens with EPHA1 and EPHA2 being generally diminished in tumors compared to normal renal tissue, whereas EFNA1 was commonly elevated. A positive EPHA1 and EPHA2 protein staining as well as a low EFNA1 protein level were significantly linked to more aggressive tumor features, but only a positive EPHA1 immunoreactivity was significantly associated with poor survival. In subgroup analyses, EPHA1 and EPHA2 protein levels were significantly higher in metastatic than in primary lesions. Patients with EPHA1/EPHA2-positive tumors or with tumors with positive EPHA1 and low EFNA1 immunoreactivity had the shortest survival rates compared to the respective other combinations. In a multivariate model, EPHA1 was an independent prognostic marker for different survival endpoints. In conclusion, an impaired EPH-ephrin signaling could contribute to the pathogenesis and progression of clear cell renal cell carcinoma.

## Introduction

About 90% of renal malignancies are renal cell carcinomas (RCC) with clear cell RCC (ccRCC) being the most common histological subtype [1]. Due to its high potential to metastasize, RCC is the urologic tumor entity with the highest mortality rate [2]. In fact, 10–20% of the cases present metastasis at the time of diagnosis and about 20–30% develop metastasis in the follow-up time [2]–[4]. The most common sites of metastatic spread in RCC are lung, bone, adrenal gland, liver and brain, whereupon more than one organ system is often involved in the metastatic process [3]. The prognosis of metastasized RCC has been improved with the new targeted therapies, but remains unfavorable as most patients develop a therapy resistance [5]. The routinely estimation of prognosis is conducted according to the TNM staging (UICC 2010) and Fuhrman grading [1]. However, recent research focuses on the identification of molecular factors which could serve as prognostic markers in addition to the clinical parameters. Despite numerous studies no reliable molecular prognostic markers in RCC have been identified to date [6].

Previously, we have identified DNA copy number abnormalities in the chromosomal region 7q11.21-7qter in 32% of the analyzed ccRCC [7]. This chromosomal region encodes among others the gene of the ephrin receptor A1 (*EPHA1*). Ephrin receptors (EPH) are the largest subfamily of transmembrane receptor tyrosine kinases (RTKs) that bind membrane-bound ligands, the ephrins. The EPH–ephrin-complexes emanate their signals in a bidirectional manner into the adjacent cells followed by internalization and degradation of the complexes [8]. EPH-ephrin signaling is a critical mediator of angiogenesis and furthermore, involved in the regulation of cell morphology, growth, migration, adhesion, and survival [8], [9]. EPH receptors and ephrins are differentially expressed in a variety of human malignant tumors and an imbalance in the receptor-ligand-ratio or an impaired receptor-ligand-interaction can affect the cellular behavior of cancer cells *in vitro* and *in vivo* [8]. Depending on the tumor type and context EPH-ephrin signaling can suppress tumor progression or promote cancer growth [8]. For instance, EPHA2 can mediate ligand-dependent inhibition and ligand-independent stimulation of cell migration and invasion [10].

Over-expression of EPHA2 in tandem with a diminished engagement with the EFNA1 ligand can lead to increased motility and invasive properties of tumor cells which is consistent with a pro-metastatic phenotype [10]. An over-expression of EPHA2 has been observed in several tumor entities, which in turn was often linked to more aggressive tumor features and/or worse prognosis [11]–[17]. A differential expression in tumors of various origins has also been shown for EPHA1 [18]–[20] and EFNA1 [11], [12], [14], [17].

In a very small sample setting, mRNA of *EPHA1*, *EPHA2* and their ligand *EFNA1* has been detected in normal (n = 3) and malignant (n = 2) kidney tissues [21]. Herrem *et al.* investigated the protein expression of EPHA2 in a small RCC cohort with mixed histological subtypes including 30 ccRCC and four non-ccRCC, whereupon EPHA2 protein levels inversely correlated with progression-free interval and overall survival period [22]. However, extensive studies on the expression and prognostic relevance of EPHA1, EPHA2 and EFNA1 in ccRCC are not available to date. Therefore, the aim of this study was to evaluate the mRNA and protein expression of these factors and to investigate their prognostic relevance in ccRCC.

## Materials and Methods

### Tissue specimens

Tissue collection and analysis was approved by the internal review board of the TU Dresden (EK194092004, EK195092004, and EK142042011). Written informed consent was obtained from each patient. All cases included in this study underwent nephrectomy for ccRCC between 1993 and 2006. Tissue microarrays (TMAs) were constructed using formalin-fixed paraffin-embedded tissue of primary tumors (one to seven cores per case) and corresponding non-malignant tissues (one to three cores per case) of 241 ccRCC patients. The TMAs contained also at least one core of resected metastases from 73 ccRCC patients. Fresh-frozen primary ccRCC samples and matched non-malignant tissue samples from 75 patients were used for gene expression analyses. Serial cryosections of available tissues were prepared and the tumor cell amount was estimated by an experienced pathologist (M.T.) on the hematoxylin-eosin stained serial tissue sections. The tumor cell amount of the ccRCC cases was at least 70% and that of the matched non-malignant specimens less than 10%. All tumors were reevaluated, staged according to the UICC 2010 classification and graded according to the Fuhrman grading system. The demographic and clinicopathological data of the patients from both sample cohorts are summarized in **Table 1**.

**Table 1:** Demographic and clinicopathological characteristics of patients included in the study.

Parameter	Immunohistochemical analyses	Gene expression analyses
**Patients (n)**	241	75
**Age at nephrectomy (years)**		
Median (Range)	62.5 (32–88)	63.4 (32–88)
**Gender (n/%)**		
female	81/34%	31/41%
male	160/66%	44/59%
**pT stage (n/%)**		
pT1/2	160/66%	58/77%
pT3/4	81/34%	17/23%
**Lymph node status (n/%)** 1		
pN0/N0	214/89%	70/93%
N1	27/11%	5/7%
**Distant metastases (n/%)**		
M0	200/83%	75/100%
M1	40/16.6%	
Unknown	1/0.4%	
**Grade (n/%)**		
G1/2	138/57.3%	41/55%
G3/4	102/42.3%	34/45%
Unknown	1/0.4%	
**Progression status (n/%)**		
No	112/47%	54/72%
Yes	68/28%	19/25%
Unknown	18/7%	
Excluded^2^	43/18%	2/3%
Follow-up: Median (Range) (months)	88 (2–222)	101 (8–173)
Time to progression: Median (Range) (months)	25.5 (5–141)	28 (8–129)
**Survival status (n/%)**		
Alive	130/54%	46/61%
Died of ccRCC	73/30%	17/23%
Died of other or unknown causes	34/14%	12/16%
Unknown	4/2%	
Follow-up: Median (Range) (months)	89 (0–222)	106 (9–173)
Time to death of ccRCC: Median (Range) (months)	40 (0–166)	45 (9–147)
Time to death of any cause: Median (Range) (months)	45 (0–188)	45 (9–154)

1 When no clinical (N0) or pathological (pN0) lymph node metastases were noticed the lymph node status was considered as pN0/N0.

2 Patients with a time to progression of ≤3 months, distant metastases (M1) or unknown M stage at nephrectomy have been excluded from analysis of progression-free survival.

### Isolation and reverse transcription of RNA

Total RNA was isolated from sections of fresh-frozen malignant (tumor cell amount ≥70%) and matched non-malignant (tumor cell amount ≤10%) tissue samples using the Invisorb Spin Tissue RNA Mini Kit (Stratec Molecular, Berlin, Germany) according to the manufacturer's instructions. RNA quality and quantity was determined with the Bioanalyzer 2100 (Agilent Technologies, Böblingen, Germany). Up to 500 ng total RNA were reverse transcribed into cDNA using SuperScript II Reverse Transcriptase (200 U; Life Technologies, Darmstadt, Germany), dNTP mix (10 pmol of each dNTP; GE Healthcare, Freiburg, Germany) and random hexamer primers (200 ng, GE Healthcare) according to manufacturers' recommendations.

### Analysis of gene expression

*EPHA1*, *EPHA2* and *EFNA1* transcript levels were measured by quantitative polymerase chain reaction (qPCR) using the LightCycler 480 system (Roche, Mannheim, Germany) in a 96-well plate format. *PPIA* (peptidylprolyl isomerase A) served as reference gene. By using gene-specific TaqMan Gene Expression Assays (*EPHA1*: Hs00975879_m1, *EPHA2*: Hs00171656_m1, *EFNA1*: Hs00358886_m1, *PPIA*: Hs99999904_m1) and the TaqMan Gene Expression Master Mix (all from Life Technologies) PCR amplification was performed in a total reaction volume of 20 µl according to the manufacturer's instructions. Crossing points (CP) were measured within two independent experiments (mean deviation ≤0.25 CP) and the mean value was used for further calculations. Standard curves were used to determine the transcript number of a single gene. Relative gene expression levels were obtained by normalization to the reference gene *PPIA*. Fold expressions were then determined as ratio of the median relative expression values of either gene in malignant to non-malignant renal tissues.

### Immunohistochemistry

TMA sections (2 µm) were deparaffinized and immersed in 0.3% hydrogen peroxide for 10 min to block endogenous peroxidase activity. The staining was performed automatically on the BenchMark XT (Ventana/Roche, Mannheim, Germany). The antigen retrieval was done for 90 min with cell conditioner buffer (Ventana). Thereafter, the slides were incubated with the primary polyclonal antibodies against EPHA1 (1∶10; Clone ab5376, Abcam, Cambridge, UK), EPHA2 (1∶30; Clone ab5386, Abcam) or EFNA1 (1∶50; Clone NBP1-30503, Novus Biologicals, Littleton, CO, USA) followed by incubation with the amplification kit and detection system Ultra View Universal (Ventana). Staining was visualized with diaminobenzidine solution followed by counterstaining with hematoxylin. To ascertain sensitivity and specificity, primary antibodies were omitted on control sections. Immunohistochemistry staining was evaluated by an experienced pathologist (M.T.) blinded to the clinicopathological parameters and the clinical outcome of the patients.

The most cases with positive staining for EPHA1 and EPHA2 were weakly to moderately positive, so we categorized the cases into negative or positive for EPHA1 and EPHA2. EFNA1 positive staining varied from weak to moderate and strong, so we grouped the cases in low EFNA1 expression (negative or weakly positive) and high EFNA1 expression (moderately to strongly positive). Since tumor cells stained uniformly across the samples the percentage of tumor cells with positive staining was not considered for statistical calculations. For each case, all evaluable cores were analyzed and then averaged. The number of evaluable cores differed for the individual proteins due to staining artifacts or loss of tissue cores during processing of the TMA slides.

### Statistical analysis

Statistical analyses were carried out with the IBM SPSS Statistics 21.0.0.0 software (IBM, Ehningen, Germany). The Mann–Whitney *U* test was used for two group comparisons. Associations of categorial variables were evaluated using the chi-squared test. For multiple comparisons p values were corrected using the method of Benjamini and Hochberg to control the false discovery rate, which is suitable for explorative studies [23].

Survival rates and median survival times were determined by the Kaplan-Meier method and differences in the survival rates between groups were compared using the logrank test. Five-year and ten-year survival rates were determined using life tables. Univariate and multivariate (stepwise forward-inclusion) Cox regression analyses were performed to identify prognostic factors for the different survival endpoints. Follow-up of patients was retrieved from in-house medical records and from the Regional Clinical Cancer Registry (Dresden, Germany). For gene expression results patients were assorted according to their median transcript levels to define an appropriate grouping (≤median *vs* >median). For EPHA1 and EPHA2 protein expression patients were classified into categories of negative and positive expression. For EFNA1 protein expression patients were categorized into low and high expression. Clinicopathological parameters were dichotomized as indicated.

## Results

### *EPHA1*, *EPHA2* and *EFNA1* mRNA expression in ccRCC and matched normal tissue

Compared to matched normal tissue, the median mRNA expression of *EPHA1* was significantly lower (6.9-fold) and that of *EFNA1* was significantly higher (1.6-fold) in ccRCC tissue specimens, while the median mRNA expression of *EPHA2* was not significantly altered (**Table S1**). Furthermore, *EPHA1* was down-regulated in about 91% of the tumors, whereas *EFNA1* was up-regulated in about 55% of the tumors (**Table S1**). The level of the mRNA expression of *EPHA1*, *EPHA2* and *EFNA1* showed no significant associations with clinicopathological parameters and survival (data not shown).

### EPHA1, EPHA2 and EFNA1 protein expression in ccRCC and matched normal tissue

**Figure 1:**
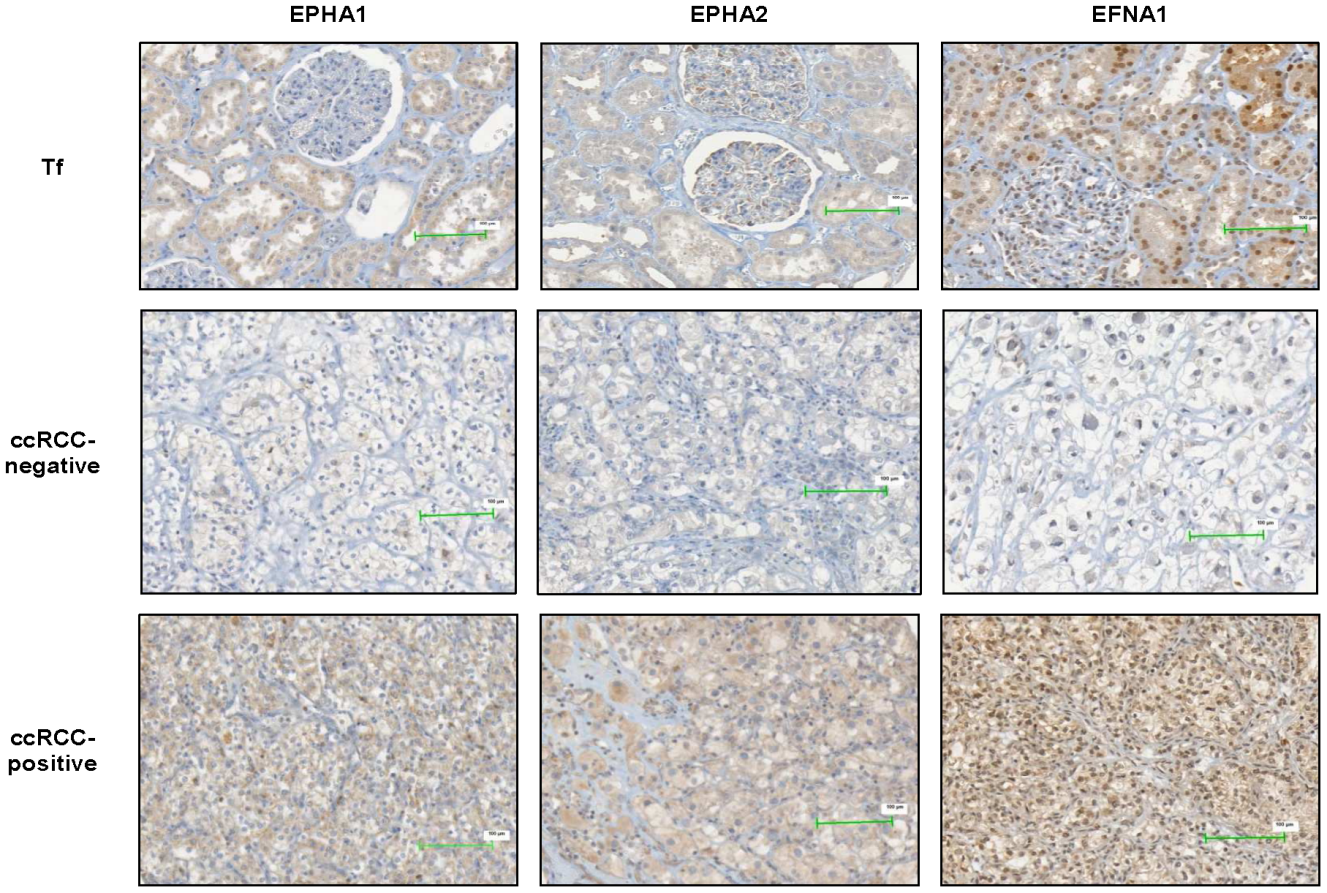
Representative images of immunohistochemically determined protein expression in non-malignant renal tissue (Tf) and ccRCC. The scale bar is 100 µm. Abbreviations: Tf: tumor-free normal kidney specimens.

For EPHA1 and EPHA2 a weak to moderate cytoplasmic expression was detected in the tubuli and in mesangial cells of the glomeruli in normal kidney tissue (**Figure 1**). Compared to non-malignant kidney tissue the protein expression of EPHA1 and EPHA2 was generally lower in tumors (**Figure 1**). Among the evaluable ccRCC specimens, 120 of 226 (53.1%) and 171 of 230 (74.3%) were negative for EPHA1 and EPHA2, respectively. Weak to moderate positive EPHA1 and EPHA2 protein expression was observed in 106 (46.9%) and 59 (25.7%) specimens, respectively.

EFNA1 showed a weak to moderate nuclear and cytoplasmic positivity in the tubuli. Tumor cells exhibited cytoplasmic and nuclear staining for EFNA1 which was observed in all positive cells. The protein expression of EFNA1 was generally higher in tumors compared to normal renal tissue. Only 56 of 206 (27.2%) tumor cases were negative for EFNA1, whereas 91 (44.2%) demonstrated a weak and 59 (28.6%) a moderate to strong positivity for EFNA1 (**Figure 1**).

A positive EPHA1 protein expression was significantly associated with a positive lymph node status and poorly differentiated tumors, whereas a positive EPHA2 protein expression was significantly related to the presence of distant metastases (**Table 2**). The EFNA1 protein level was significantly linked to tumor stage, whereupon patients with advanced tumor stage frequently showed a low EFNA1 protein expression (**Table 2**).

**Table 2:** Association between EPHA1, EPHA2 and EFNA protein expression in primary tumors and clinicopathological parameters in patients with ccRCC.

	EPHA1		EPHA2		EFNA1	
Parameter	negative (n = 120)	positive (n = 106)	negative (n = 171)	positive (n = 59)	low (n = 147)	high (n = 59)
**pT stage**	p = 0.071		p = 0.879		**p = 0.004**	
pT1/2	84 (57.5%)	62 (42.5%)	112 (74.7%)	38 (25.3%)	86 (64.7%)	47 (35.1%)
pT3/4	36 (45.0%	44 (55.0%)	59 (73.8%)	21 (26.3%)	60 (83.3%)	12 (16.7%)
**Lymph node status** 1	**p = 0.015**		p = 0.039		p = 0.167	
pN0/N0	112 (56.0%)	88 (44.0%)	156 (76.5%)	48 (23.5%)	127 (69.8%)	55 (30.2%)
pN1	8 (30.8%)	18 (69.2%)	15 (57.7%)	11 (42.3%)	20 (83.3%)	4 (16.7%)
**Distant metastases** 2	p = 0.072		**p = 0.008**		p = 0.595	
M0	103 (55.7%)	82 (44.3%)	147 (77.8%)	42 (22.2%)	120 (70.6%)	50 (29.4%)
M1	16 (40.0%)	24 (60.0%)	23 (57.5)	17 (42.5%)	27 (75.0%)	9 (25.0%)
**Grade**	**p<0.001**		p = 0.213		p = 0.225	
G1/2	82 (64.6%)	45 (35.4%)	100 (77.5%)	29 (22.5%)	76 (67.9%)	36 (32.1%)
G3/4	38 (38.4%)	61 (61.6%)	71 (70.3%)	30 (29.7%)	71 (75.5%)	23 (24.5%)

The chi-squared test was used to evaluate the associations of the categorized variables. The p values were then corrected for multiple comparisons (n = 12) by using the method of Benjamini and Hochberg. P values highlighted in bold indicate statistically significant associations.

1 When no clinical (N0) or pathological (pN0) lymph node metastases were noticed the lymph node status was considered as pN0/N0.

2 For M status only 225 (EPHA1) and 229 (EPHA2) cases, respectively, were available due to one case with unknown M status.

### EPHA1, EPHA2 and EFNA1 protein expression in metastases compared to primary tumors

**Figure 2:**
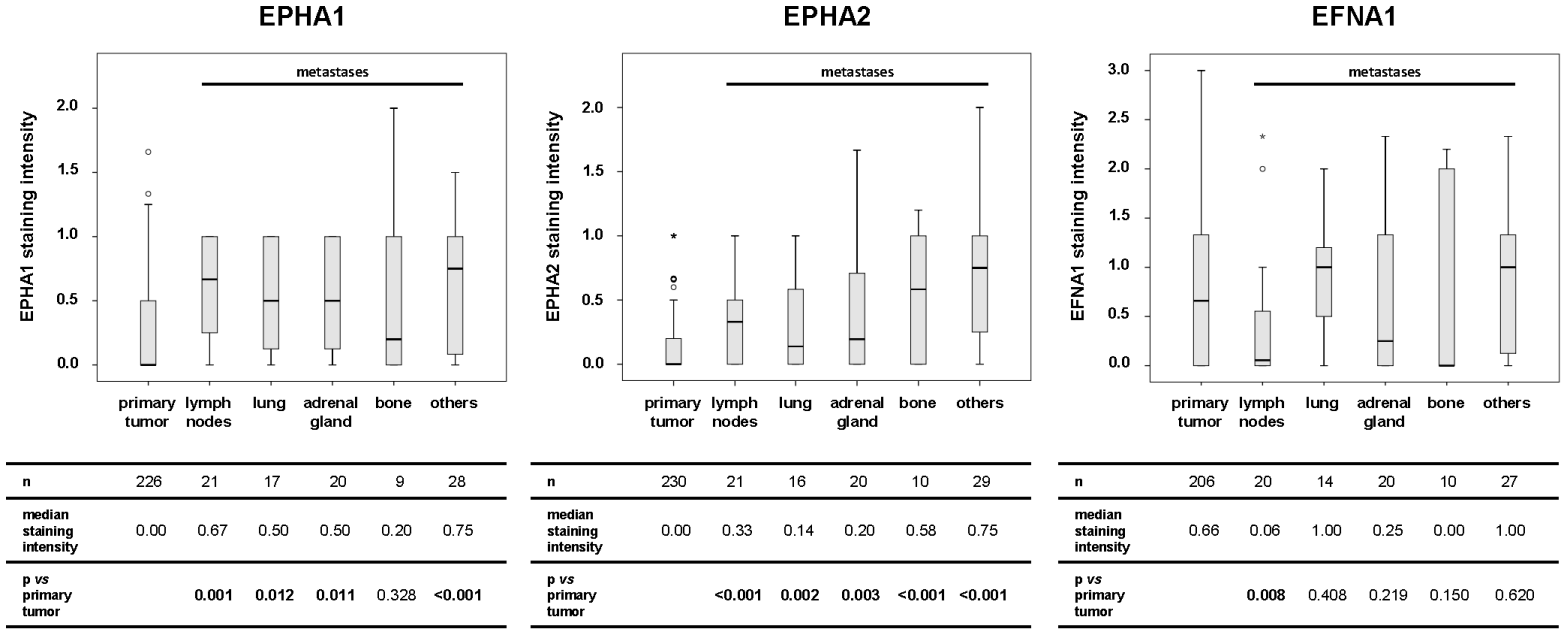
Differential protein expression of EPHA1, EPHA2 and EFNA1 in primary tumors and subgroups of metastases. The table beneath each image includes the number of patients and the median staining intensity for each group as well as the p values retrieved by the Mann–Whitney *U* test. P values highlighted in bold indicate statistically significant differences following corrections for multiple comparisons (n = 5 for each protein) by using the method of Benjamini and Hochberg.

The protein expression of EPHA1, EPHA2 and EFNA1 in primary tumors was compared to the protein expression of ccRCC metastases in different metastatic sites (lymph nodes, lung, adrenal gland, bone, others) (**Figure 2**). EPHA1 protein expression was significantly elevated in all metastases except for bone metastases, whereas EPHA2 protein expression was significantly increased in all subgroups of metastases compared with primary ccRCC. Compared to the primary tumor, the EFNA1 expression was lower in metastases to the lymph nodes, adrenal gland, and bone. This diminished expression was only significant for lymph node metastases. Furthermore, statistical significance regarding the expression of the three factors in the respective metastases subgroups was mostly retained when compared only to the matched primary tumors (n = 73 for EPHA1 & EPHA2, n = 68 for EFNA1; data not shown).

### Influence of clinicopathological parameters and molecular markers on survival

As expected, patients with more aggressive tumor features had mostly a significantly shorter progression-free survival (PFS), tumor-specific survival (TSS) and overall survival (OS) which was reflected by lower five-year and ten-year survival rates (**Table S2**). Univariate Cox analysis regarding the predictive value of the clinicopathological parameters also confirmed these results (**Table 3**).

**Table 3:** Univariate Cox regression analyses for PFS, TSS and OS dependent on clinicopathological parameters and molecular markers.

	PFS			TSS			OS		
Parameter	HR	95% CI	p value	HR	95% CI	p value	HR	95% CI	p value
pT stage (pT3/4 *vs* pT1/2)	2.86	1.75–4.66	**<0.001**	2.67	1.68–4.23	**<0.001**	2.31	1.57–3.38	**<0.001**
N stage (pN1 *vs* pN0/N0)	16.04	7.80–32.99	**<0.001**	9.60	5.54–16.66	**<0.001**	7.13	4.37–11.64	**<0.001**
M stage (M1 *vs* M0)	n.d.			6.48	3.96–10.61	**<0.001**	4.66	3.00–7.22	**<0.001**
Grade (G3/4 *vs* G1/2)	1.40	0.86–2.26	0.174	2.02	1.27–3.21	**0.003**	1.97	1.34–2.89	**0.001**
EPHA1 protein (positive *vs* negative)	2.10	1.29–3.41	**0.003**	2.04	1.27–3.26	**0.003**	1.82	1.23–2.67	**0.003**
EPHA2 protein (positive *vs* negative)	1.14	0.64–2.02	0.660	1.61	0.97–2.68	0.068	1.56	1.01–2.40	0.043
EFNA1 protein (high *vs* low)	0.55	0.30–1.02	0.056	0.76	0.43–1.34	0.347	0.68	0.42–1.11	0.122

P values highlighted in bold indicate statistically significant prognostic markers following correction for multiple comparisons (n = 20) by using the method of Benjamini and Hochberg.

Abbreviations: CI: confidence interval; HR: hazard ratio; n.d.: not determined.

**Figure 3:**
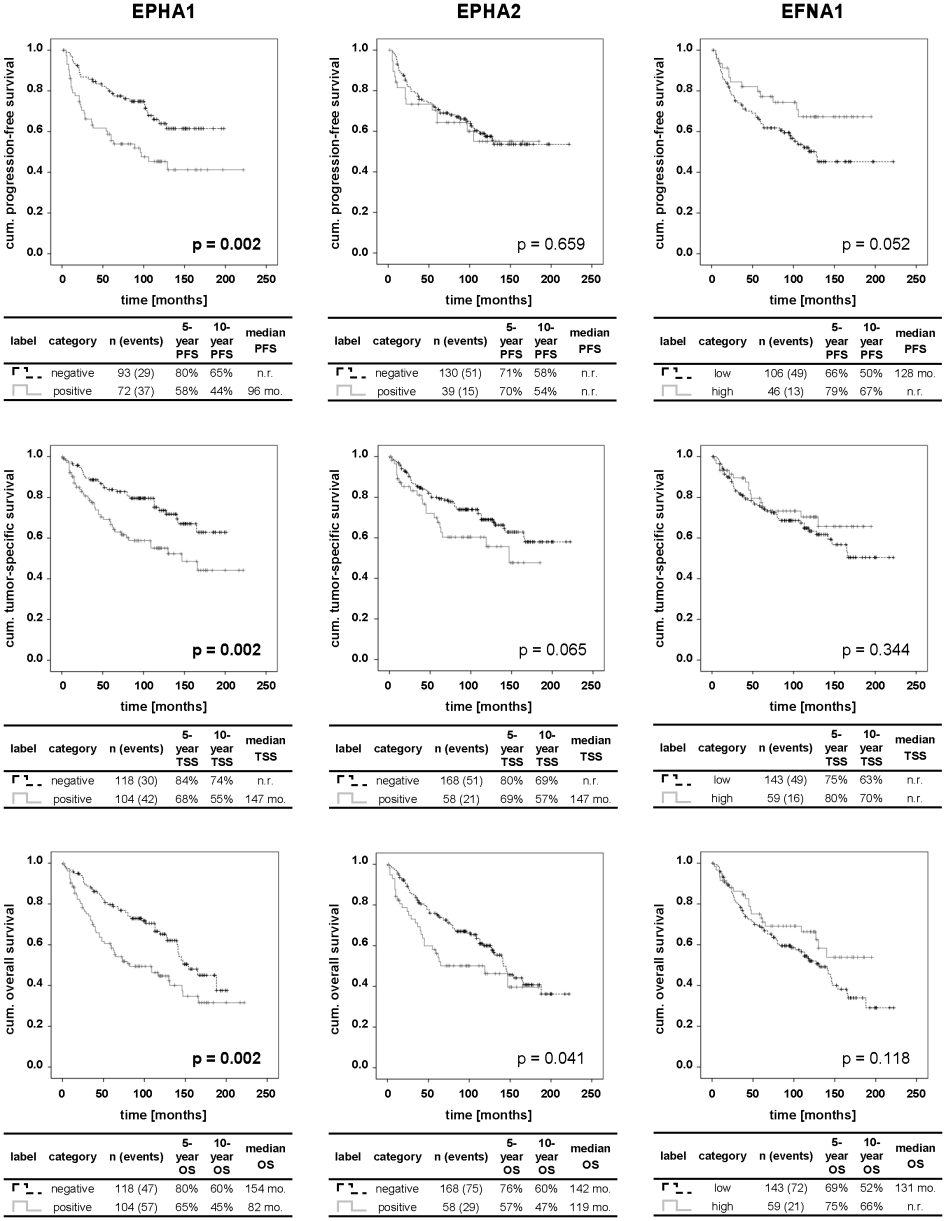
Kaplan-Meier analysis of PFS, TSS and OS of ccRCC patients dependent on protein expression. The table beneath each Kaplan-Meier curve includes the legend, the number of patients and events in each category as well as the respective median survival times, 5- and 10-year survival rates. P values were calculated by logrank test and then corrected for multiple comparisons (n = 29) by using the method of Benjamini and Hochberg. P values highlighted in bold indicate statistically significant differences. Abbreviations: cum.: cumulative; mo.: months; n.r.: not reached.

Intriguingly, a lack of EPHA1 protein expression was significantly associated with a longer PFS, TSS and OS which was mirrored by respective higher five-year and ten-year survival rates than for cases with a detectable EPHA1 protein expression (**Figure 3**). These results were also confirmed in univariate Cox analysis (**Table 3**). In contrast, neither EPHA2 nor EFNA1 protein expression showed a significant association to various survival endpoints in ccRCC patients (**Figure 3**, **Table 3**).

Next, the prognostic relevance of the individual markers was investigated in a multivariate model with a stepwise forward-inclusion of the clinicopathological parameters and molecular markers (**Table 4**). For the molecular markers, only EPHA1 protein expression was an independent prognostic factor for all three survival endpoints.

**Table 4:** Multivariate Cox regression analyses for PFS, TSS and OS dependent on clinicopathological parameters and molecular markers.

	PFS			TSS			OS		
Parameter	HR	95% CI	p value	HR	95% CI	p value	HR	95% CI	p value
pT stage (pT3/4 *vs* pT1/2)	2.080	1.193–3.629	**0.010**			n.s.			n.s.
N stage (pN1 *vs* pN0/N0)	9.825	4.316–22.368	**<0.001**	7.771	4.230–14.275	**<0.001**	5.722	3.333–9.824	**<0.001**
M stage (M1 *vs* M0)	n.i.			6.125	3.591–10.444	**<0.001**	4.304	2.669–6.942	**<0.001**
Grade (G3/4 *vs* G1/2)			n.s.	1.766	1.029–3.031	**0.039**	1.787	1.148–2.781	**0.010**
EPHA1 protein (positive *vs* negative)	1.776	1.054–2.992	**0.031**	2.035	1.179–3.514	**0.011**	1.721	1.110–2.669	**0.015**
EPHA2 protein (positive *vs* negative)			n.s.			n.s.			n.s.
EFNA1 protein (high *vs* low)			n.s.			n.s.			n.s.

For multivariate Cox analysis, clinicopathological parameters and protein markers were included stepwise resulting in a final model which only included variables with significance levels <0.05. P values highlighted in bold indicate statistically significant and independent prognostic markers.

Abbreviations: CI: confidence interval; HR: hazard ratio; n.i.: not included; n.s.: not significant.

### Combined influence of molecular markers on survival

**Figure 4:**
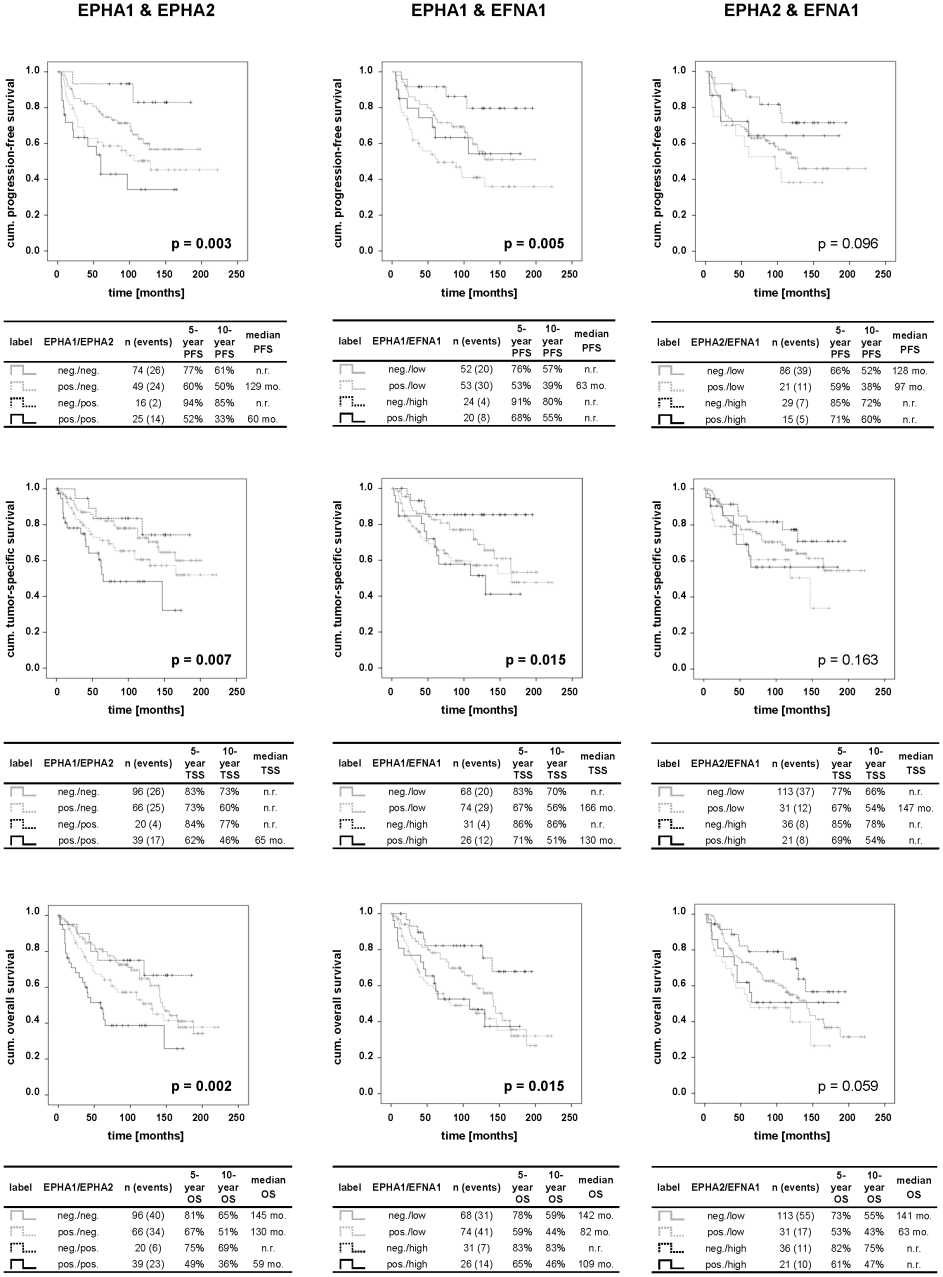
Kaplan-Meier analysis of PFS, TSS and OS of ccRCC patients dependent on pairwise-combined protein expression. The table beneath each Kaplan-Meier curve includes the legend, the number of patients and events in each category as well as the respective median survival times, 5- and 10-year survival rates. P values were calculated by logrank test and then corrected for multiple comparisons (n = 29) by using the method of Benjamini and Hochberg. P values highlighted in bold indicate statistically significant differences. Abbreviations: cum.: cumulative; mo.: months; neg.: negative; n.r.: not reached; pos.: positive.

Next, we evaluated the combined influence of the three factors on the different survival endpoints (**Figure 4**). A positive protein expression of both EPHA1 and EPHA2 was generally linked to a shorter survival than negative expression of both receptors and cases positive for only one receptor (EPHA1 or EPHA2). Furthermore, a positive expression of EPHA1 protein combined with low EFNA1 protein levels was significantly associated with a shorter survival particularly compared to patients exhibiting a negative EPHA1 and a high EFNA1 immunoreactivity. The combination of EPHA2 with EFNA1 did not show any significant influence on the various survival endpoints of ccRCC patients.

## Discussion

RTKs of the EPH family and their ephrin ligands are known to play an important role in the regulation of cell morphology, growth, migration, adhesion, and survival as well as in angiogenesis [8], [9]. An abnormal expression of EPHA1, EPHA2 and EFNA1 with influence on patient outcome has been demonstrated in different tumor entities [11]–[17], [19], [24], [25]. In small sample cohorts, these factors have also been shown to be differentially expressed in normal and malignant renal tissue [21], [22]. However, the mRNA and protein expression patterns of EPHA1, EPHA2 and EFNA1 have not been systematically studied in ccRCC yet, and the prognostic relevance of these factors in ccRCC is still unclear. In a previous study, we have shown that 32% of the analyzed ccRCC exhibited aberrations in the chromosomal region containing the *EPHA1* gene [7]. Therefore, the EPH signaling pathway could be of functional relevance in ccRCC which motivated us to investigate the expression of EPHA1, EPHA2 and EFNA1 in a large, well-characterized ccRCC patient cohort.

The EPHA1 expression was diminished both at the mRNA and protein level in a high percentage of ccRCC. EPHA1 protein levels showed significant associations with clinicopathological parameters, whereupon patients with an absent EPHA1 immunoreactivity frequently had a lower grade (p<0.001) and absent lymph node metastases (p = 0.015). This is in line with studies in esophageal [20] and gastric cancers [19], where a lower EPHA1 protein expression was significantly associated with less aggressive tumor features like lower tumor stage, absence of lymph node metastases and lower grade. The present study further demonstrated that ccRCC cases lacking EPHA1 protein exhibited a significantly longer survival than ccRCC patients expressing EPHA1. In a multivariate analysis the lack of EPHA1 expression emerged as an independent prognostic factor for PFS, TSS and OS in ccRCC. This is the first study to demonstrate the potential usefulness of EPHA1 protein staining for ccRCC prognosis. In line with our results, a longer survival was also reported for gastric carcinoma patients with low EPHA1 protein levels [19]. In lung cancer, no associations between EPHA1 and clinicopathological features or survival were noticed [26]. Contrary to our study, a lower EPHA1 protein expression was linked to aggressive tumor features and shorter survival in colorectal carcinomas [18]. This suggests that the role of EPHA1 in tumorigenesis and metastasis depends on the tumor entity.

The other EPH receptor included in our study, EPHA2, showed no significant differences in the mRNA expression between ccRCC and corresponding non-malignant tissue. At the protein level, ccRCC showed a lower expression of EPHA2 compared with benign renal tissue. A lack of EPHA2 protein expression was significantly linked to the absence of distant metastases (p = 0.008), but not to survival. In a small cohort of 34 RCC, which also included four non-ccRCCs, Herrem *et al.* demonstrated that EPHA2 protein expression is elevated in high-grade tumors and correlated inversely with progression-free interval and overall survival period [22]. Although we could not find an association between EPHA2 and survival based on Kaplan-Meier and univariate Cox regression analysis, the results by Herrem *et al.* and ours suggest that EPHA2 expression may be correlated with more aggressive tumor features in ccRCC. In other solid tumors like vulvar [12], cervical [11], endometrial [13], [16], gastric [17] and head/neck [15] carcinomas, higher EPHA2 protein levels were also associated with advanced disease stages and/or poor survival.

Furthermore, the mRNA expression and immunohistochemical staining of EFNA1, which is the ligand to both EPHA1 and EPHA2, was generally higher in ccRCC specimens compared to normal renal tissue. High levels of EFNA1 protein have also been reported in vulvar [12], cervical [11] and gastric [17] carcinomas being associated with advanced and aggressive tumors. Contrary, in the present study, ccRCC patients with a high EFNA1 protein expression had frequently a lower tumor stage (p = 0.004). We did not find any link between EFNA1 protein expression and patient outcome, which is supported by other studies in patients with gastric [17] and lung [27] cancer. However, a high protein expression of EFNA1 was linked to poor survival in patients with vulvar [12] and cervical [11] carcinomas.

In addition to their altered protein expression in primary ccRCC, all three molecular markers were differentially expressed in ccRCC metastases of different secondary sites. Particularly, EPHA1 and EPHA2 protein levels were higher in all subgroups of ccRCC metastases than in primary tumors. In line with this, Tatsumi *et al.* demonstrated that EPHA2 protein is expressed at higher levels in metastatic ccRCC cell lines than in primary ccRCC cell lines [28]. Furthermore, EPHA2 protein expression in brain metastases was significantly higher compared to matched primary lung cancers (n = 10) [29].

Furthermore, we could demonstrate a combined influence of the molecular markers on survival. Particularly, the pairwise combination of EPHA1 with either EPHA2 or EFNA1 displayed a significant influence on survival. For that matter, patients with EPHA1/EPHA2-positive tumors and with tumors with positive EPHA1 and low EFNA1 immunoreactivity had the shortest survival rates compared to the respective other combinations. These findings further highlight the important role of the interplay between EPH receptors and ligands in cancer progression. In malignant gliomas, patients positive for EPHA2 and negative for EFNA1 protein exhibited the shortest survival compared to other patients [14]. This might be explained by the pro-oncogenic function of EPHA2 in the absence of EFNA1 [10], whereas activation of EPHA2 by EFNA1 promoted tumor suppression by triggering receptor degradation [30]. However, in the present study, EPHA1 over-expression particularly in combination with low EFNA1 levels and/or EPHA2 over-expression seem to be the dominating influence on ccRCC progression. How EPHA1 acts is not definitely elucidated, but the ratio of EPH receptors to ligands is thought to be an important determinant of tumor progression [8].

In addition to tumor progression and metastasis, EPH-ephrin signaling is also a critical mediator of tumor angiogenesis [8], [9]. For instance, EFNA1-mediated EPHA2 activation is required for maximal neoangiogenesis by VEGF *in vivo* [31]. To date, functional reports on characterizing the role of EPHA1 in tumor angiogenesis are still missing. Nevertheless, inhibition of EPHA1 [32] and EPHA2 [14], [16], [33], [34] resulted in reduced tumor growth and invasiveness in various oncogenic animal models. This anti-tumor efficacy was often accompanied by an inhibition of angiogenesis [16], [32], [33] which would be an advantage in the therapy of highly angiogenic cancers such as ccRCC.

Our findings indicate a functional role for the investigated EPH/ephrin signaling factors in ccRCC progression with particular emphasis on EPHA1. One could speculate that tumor cells with an “aggressive” expression status, i.e. over-expression of EPHA1 and EPHA2 as well as down-regulation of EFNA1, possess a survival advantage under adverse conditions and thus, are capable of thriving in the foreign microenvironment of secondary sites. This hypothesis is further supported by recent evidence showing that EPHA2-expressing prostate cancer cells gain an invasive advantage which was crucial to successfully colonize distant organs [35]. Based on the results that EPHA1, EPHA2 and EFNA1 are differentially expressed in metastatic compared to primary tissue, the EPH kinases and/or their ligands represent attractive candidates for a targeted treatment of metastatic ccRCC.

Taken together, the EPH receptors EPHA1 and EPHA2 as well as their ligand EFNA1 could play an important role in ccRCC initiation and progression. The present results further indicate that particularly EPHA1 may be useful as a prognostic marker for ccRCC as the absence of EPHA1 protein expression is a favorable independent prognosticator. Ultimately, a modulation of the EPH-ephrin signaling pathway could represent an alternative therapeutic strategy for ccRCC patients. Nevertheless, further research is needed to understand the precise involvement of EPHA1, EPHA2 and EFNA1 in ccRCC initiation and progression.

## Supporting Information

Table S1 **Differential mRNA expression of** ***EPHA1*** **,** ***EPHA2*** **and** ***EFNA1*** **in ccRCC and matched non-malignant kidney tissues samples.** A fold expression of ≥1.5 was considered as up-regulation and of ≤−1.5 as down-regulation, whereas the remaining fraction was regarded as an unaltered expression. A Mann–Whitney *U* test was performed to assess whether there is a statistical difference between expression levels in ccRCC and Tf samples. P values highlighted in bold indicate statistically significant differences. ^1^ Due to loss of tissue during processing only 74 Tf tissue specimens were available for the mRNA expression analysis of *EPHA2*.
(DOC)

Table S2 **Median survival times, 5-year and 10-year survival rates as well as p values retrieved by the logrank test from the Kaplan-Meier survival analysis for PFS, TSS and OS dependent on clinicopathological parameters.** P values highlighted in bold indicate statistically significant differences following correction for multiple comparisons (n = 29) by using the method of Benjamini and Hochberg. ^1^ When no clinical (N0) or pathological (pN0) lymph node metastases were noticed the lymph node status was considered as pN0/N0.
(DOCX)
