# Supplementary material for: Lack of Ephrin Receptor A1 Is a Favorable Independent Prognostic Factor in Clear Cell Renal Cell Carcinoma
Source: PLoS One. 2014 Jul 15;9(7):e102262. doi: 10.1371/journal.pone.0102262 (PMC4099180; doi:10.1371/journal.pone.0102262)
Supplement: Table S1 — Differential mRNA expression of EPHA1 , EPHA2 and EFNA1 in ccRCC and matched non-malignant kidney tissues samples. A fold expression of ≥1.5 was considered as up-regulation and of ≤−1.5 as down-regulation, whereas the remaining fraction was regarded as an unaltered expression. A Mann–Whitney U test was performed to assess whether there is a statistical difference between expression levels in ccRCC and Tf samples. P values highlighted in bold indicate statistically significant differences. 1 Due to loss of tissue during processing only 74 Tf tissue specimens were available for the mRNA expression analysis of EPHA2. (DOC) [file pone.0102262.s001.doc]

**Supporting Information Table S1: Differential mRNA expression of *EPHA1*, *EPHA2* and *EFNA1* in ccRCC and matched non-malignant kidney tissues samples.**

|  | ***EPHA1*** | ***EPHA2*** | ***EFNA1*** |
| --- | --- | --- | --- |
| **Relative median transcript levels (x 10^-3^)** |  |  |  |
| ccRCC | 0.96 | 24.26 | 50.43 |
| Tf | 6.63 | 30.96^1^ | 31.94 |
| p value | **<0.001** | 0.108 | **<0.001** |
| **Fold expression (ccRCC/Tf_median_)** |  |  |  |
| Median fold expression | -6.9 | -1.3 | +1.6 |
| ≤ -1.5-fold down (n / %) | 68 / 90.7% | 30 / 40.0% | 14 / 18.7% |
| Unaltered (n / %) | 5 / 6.7% | 31 / 41.3% | 20 / 26.7% |
| ≥ 1.5-fold up (n / %) | 2 / 2.7% | 14 / 18.7% | 41 / 54.7% |

A fold expression of ≥1.5 was considered as up-regulation and of ≤-1.5 as down-regulation, whereas the remaining fraction was regarded as an unaltered expression. A Mann–Whitney *U* test was performed to assess whether there is a statistical difference between expression levels in ccRCC and Tf samples. P values highlighted in bold indicate statistically significant differences (p<0.05).

^1^ Due to loss of tissue during processing only 74 Tf tissue specimens were available for the mRNA expression analysis of *EPHA2*.

Abbreviations: Tf: tumor-free normal kidney specimens
