# Supplementary material for: Lack of Ephrin Receptor A1 Is a Favorable Independent Prognostic Factor in Clear Cell Renal Cell Carcinoma
Source: PLoS One. 2014 Jul 15;9(7):e102262. doi: 10.1371/journal.pone.0102262 (PMC4099180; doi:10.1371/journal.pone.0102262)
Supplement: Table S2 — Median survival times, 5-year and 10-year survival rates as well as p values retrieved by the logrank test from the Kaplan-Meier survival analysis for PFS, TSS and OS dependent on clinicopathological parameters. P values highlighted in bold indicate statistically significant differences following correction for multiple comparisons (n = 29) by using the method of Benjamini and Hochberg. 1 When no clinical (N0) or pathological (pN0) lymph node metastases were noticed the lymph node status was considered as pN0/N0. (DOCX) [file pone.0102262.s002.docx]

**Supporting Information Table S2:** Median survival times, 5-year and 10-year survival rates as well as p values retrieved by the logrank test from the Kaplan-Meier survival analysis for PFS, TSS and OS dependent on clinicopathological parameters.

|  | **PFS** | | | | **TSS** | | | | **OS** | | | |
| --- | --- | --- | --- | --- | --- | --- | --- | --- | --- | --- | --- | --- |
| **Parameter** | **n (events)** | **5-year PFS** | **10-year PFS** | **Median PFS (months)** | **n (events)** | **5-year TSS** | **10-year TSS** | **Median TSS (months)** | **n (events)** | **5-year OS** | **10-year OS** | **Median OS (months)** |
| **pT stage** | **p<0.001** | | | | **p<0.001** | | | | **p<0.001** | | | |
| pT1/2 | 137 (41) | 81% | 66% | n.r. | 158 (36) | 86% | 77% | n.r. | 158 (57) | 80% | 66% | 166 |
| pT3/4 | 43 (27) | 46% | 37% | 49 | 79 (37) | 61% | 46% | 109 | 79 (50) | 54% | 39% | 62 |
| **Lymph node status^1^** | **p<0.001** | | | | **p<0.001** | | | | **p<0.001** | | | |
| pN0/N0 | 168 (56) | 78% | 63% | n.r. | 211 (53) | 85% | 73% | n.r. | 211 (84) | 79% | 63% | 147 |
| pN1 | 12 (12) | 0% | 0% | 10 | 26 (20) | 19% | 10% | 24 | 26 (23) | 12% | 6% | 24 |
| **Distant metastases** | n.d. | | | | **p<0.001** | | | | **p<0.001** | | | |
| M0 |  |  |  |  | 198 (47) | 85% | 75% | n.r. | 198 (78) | 79% | 64% | 154 |
| M1 |  |  |  |  | 38 (26) | 35% | 20% | 34 | 38 (29) | 30% | 17% | 34 |
| **Grading** | p=0.170 | | | | **p=0.002** | | | | **p<0.001** | | | |
| G1/2 | 111 (39) | 76% | 63% | n.r. | 136 (34) | 84% | 75% | n.r. | 136 (51) | 78% | 67% | 166 |
| G3/4 | 68 (29) | 65% | 52% | n.r. | 100 (39) | 68% | 56% | n.r. | 100 (55) | 62% | 44% | 109 |

P values highlighted in bold indicate statistically significant differences following correction for multiple comparisons (n=29) by using the method of Benjamini and Hochberg.

^1^ When no clinical (N0) or pathological (pN0) lymph node metastases were noticed the lymph node status was considered as pN0/N0.

Abbreviations: n.d.: not determined; n.r.: not reached
